# Practices of Dengue Fever Prevention and the Associated Factors among the Orang Asli in Peninsular Malaysia

**DOI:** 10.1371/journal.pntd.0003954

**Published:** 2015-08-12

**Authors:** Josephine Rebecca Chandren, Li Ping Wong, Sazaly AbuBakar

**Affiliations:** 1 Department of Social and Preventive Medicine, Faculty of Medicine, University of Malaya, Kuala Lumpur, Malaysia; 2 Julius Centre University of Malaya (JCUM), University of Malaya, Kuala Lumpur, Malaysia; 3 Department of Medical Microbiology, Faculty of Medicine, University of Malaya, Kuala Lumpur, Malaysia; 4 Tropical Infectious Disease Research and Educational Centre (TIDREC), University of Malaya, Kuala Lumpur, Malaysia; Common Heritage Foundation, NIGERIA

## Abstract

**Background:**

Dengue is prevalent among Malaysia's indigenous peoples, known as the Orang Asli, and it poses a serious health threat to them. The study aims to look at the socio-demographic factors, health beliefs, and knowledge about dengue and its association to dengue prevention practices among Orang Asli communities in Peninsular Malaysia.

**Methods:**

A cross-sectional survey was conducted in 16 randomly selected Orang Asli villages from eight states in Peninsular Malaysia from April 2012 until February 2013.

**Results:**

A total of 560 Orang Asli were interviewed and 505 completed the survey. Slightly above half of the participants (n = 280, 55.4%) had a total dengue prevention score of 51–100 (of a possible score of 0–100). Multivariate analysis findings showed dengue knowledge, perceived barriers to perform dengue prevention, fogging frequency, and perceived susceptibility to dengue fever as significant factors associated to dengue prevention practices. Participants with a lower dengue knowledge score (score 0–18) were less likely (OR = 0.63, 95%CI = 0.44–0.92 vs. score 19–36, P = 0.015) to practice dengue prevention. Participants with low perceived barriers to prevent dengue (score of 1–5) were more likely (OR = 2.06, 95%CI = 1.21–3.53, vs. score of 6–10, P = 0.008) to practice dengue prevention. Villages that were not fogged (OR = 0.49, 95%CI = 0.24–0.99, P = 0.045) or rarely fogged (OR = 0.40, 95%CI = 0.22–0.75, P = 0.004) had lower dengue prevention practices than villages that were fogged often. Participants with low perceived susceptibility of acquiring dengue (score of 1–5) were less likely (OR = 0.54, 95%CI = 0.33–0.89 vs. score of 6–10, P = 0.018) to practice dengue prevention measures.

**Conclusion:**

Findings imply that educational and health programmes should focus on enhancing dengue knowledge and perceived susceptibility of acquiring dengue and reducing perceived barriers to performing dengue prevention practices among the Orang Asli. More outreach on mosquito control campaigns should be carried out especially in villages where mosquito fogging is frequent.

## Author Summary

Dengue poses a threat to everyone worldwide, including the Orang Asli community in Peninsular Malaysia. Social demographic, knowledge and behavioral factors are essential aspects to control and prevent dengue. This study is aimed to examine these factors and their association with dengue prevention practices. A cross-sectional survey was conducted among 16 randomly selected Orang Asli villages from 8 states in Peninsular Malaysia. Results showed that 1) level of dengue knowledge, 2) perceived barriers to perform dengue prevention, 3) fogging frequency, and 4) perceived susceptibility to dengue fever are significant factors of dengue prevention practices. Findings provide important insights into intervention to increase dengue prevention practices among the Orang Asli community.

## Introduction

Dengue fever is a serious problem overwhelming the world: annually, there are about 50–100 million dengue infections [1] which include 500,000 dengue hemorrhagic fever DHF cases with 22,000 deaths, mostly among children [2]. In the year 2085, is it estimated at least 50–60% of the world population will be at risk of dengue fever [3]. In Malaysia, the morbidity rate for DHF was the highest recorded from 1987 to 1991 among adults aged 20–29 years [4]. WHO recorded an increase of two times the incidence rate of dengue fever from the year 2012, with 21,900 cases, compared to year 2013, with 49,346 cases, in Malaysia.

The prevalence of dengue fever in the rural areas of Malaysia was estimated to range from 24% in the Lundu District, Sarawak [5] to about 91% throughout the Malaysian population [6]. The indigenous peoples in Malaysia, known as “Orang Asli”, make up 1% of the total population in Malaysia [7]. Approximately, 61% of the Orang Asli live at the fringes of the jungle or rural areas, while 37% live deep within the jungle, and only 1% are found in or close to urban areas. A recent study conducted in Malaysia, found that prevalence of dengue was significantly higher among the rural areas than in urban areas [8]. A study conducted in 1956 [9] among the Orang Asli population in Peninsular Malaysia showed that virtually most adults above the age of 30 years from the Temuan and Semai community in Hulu Langat, Selangor, had been affected by dengue fever. Another study conducted in 1986 [10] showed that 73% of the Temuan Orang Asli community in Kampung Tanjong Rabok, Selangor had been affected by dengue fever and its related viruses.

Human behaviour contributes majorly in controlling the breeding grounds for these mosquitoes and reducing the number of the mosquito population [11]. Vector control is one of the effective method in controlling and preventing dengue fever[12] [13]. Vector control can be done by frequent fogging in endemic areas which is mostly done outdoors. However, the *Aedes aegypti* mosquito tends to rest hidden indoors, making it hard for insecticide to reach adult mosquitoes [14]. One of the few methods of dengue prevention is eliminating the breeding sites of dengue mosquitoes indoors and outdoors. The success is accorded in Thailand where eliminating mosquito breeding has definitely reduced the number of dengue cases in the region [15]. The success of efforts in dengue prevention and control is mainly from improving public and household environmental sanitation, water supply, and alteration of human behaviour towards dengue fever [16].

Health beliefs were found to influence dengue preventive practices [17]. It is reported that the health belief model (HBM) is by far the most commonly used theory in health education and health promotion [18]. The underlying concept of the original HBM is that health behaviour is determined by personal beliefs or perceptions about a disease and the strategies available to decrease its occurrence [19] [20]. The HBM consists of four perceptions that serve as the main constructs of the model: perceived seriousness, perceived susceptibility, perceived benefits, and perceived barriers [21] and will be implemented to assess the health behaviour pattern accordingly.

It has been recognized that socio-demographic characteristics have an important impact on dengue prevention practices and control. Younger and married people reported higher prevention practices against dengue fever compared to those from older age groups and those who were single. A study conducted in Malaysia reported that eliminating breeding sites and mosquito prevention practices were higher among the Malaysian rural population compared to the aborigines [22]. This could perhaps be explained by the rural populations having a higher level of education and living nearer to health facilities. Therefore, this shows that socio-demographic characteristics are an important factor in dengue elimination and prevention.

Knowledge or awareness has been reported as important in dengue prevention and control. According to a recent study, inadequate knowledge about dengue is a major risk factor faced in the elimination of dengue [23]. A recent study found that inadequate and lack of knowledge about signs and symptoms, transmission of dengue, and preventive practices can increase the spread of dengue fever among the Malaysian population [8], [22]. A past study conducted among Malaysians found that they generally had good knowledge of dengue fever and its prevention [24]. However, evidence was found that higher knowledge did not necessarily result in adoption of the recommended preventive behaviour [25]; [26]. Therefore, further investigation is important to find out the association between knowledge and dengue prevention practices.

One of the important factors that contribute to the spread of dengue fever is the intensity of the dengue causing mosquitoes. Intensity of dengue causing mosquitoes increases when there are more available breeding sites and food. Other than that, the density of vegetation in a surrounding area is a potential habitat for *Aedes* mosquito breeding [27]. Orang Asli live in jungles and in surroundings of highly dense vegetation where many mosquitoes are found and, therefore, are at risk of diseases caused by mosquitoes. The spread of diseases and viruses caused by the mosquito is most effective in very densely populated areas [28] where it feeds almost exclusively on humans. In a study conducted among the Native Americans, the prevalence of infectious disease caused by vector was likely to be attributed to poor living conditions where house crowding with lack of sanitation is common [29]. House crowding is common among the Orang Asli as all family members live under one roof [30].

Dengue prevention practices and the associated factors have never been explored among the Orang Asli in Peninsular Malaysia. Identifying and understanding factors associated to dengue prevention may provide insight into targeted interventions to enhance dengue prevention practice and facilitate authorities in the management of dengue prevention. This study is aimed to look at these factors (socio-demographic, theoretical constructs of the HBM, and dengue knowledge) and their association with dengue fever prevention practices.

## Methods

### Sampling frame

The sample for this study were Orang Asli originating and living in Peninsular Malaysia. According to the Department of Orang Asli Development (JAKOA), there are eight states in Malaysia where Orang Asli are found. From these eight states, two villages from each state were randomly selected where JAKOA was able to provide assistance in accessing the Orang Asli of the respective states. The research group approached Orang Asli members with JAKOA's supervision to acquire better acknowledgment and responses.

Based on their location, the villages were either 1) forest fringe areas–Orang Asli villages which were relocated and have access to basic resources such as electricity and pipe water or 2) deep within the jungle–Orang Asli villages where most basic resources were not readily available.

In total, 16 Orang Asli villages were selected based on 1) accessibility of these villages by land transport and 2) permission being granted by the head of the village.

A cross-sectional study was performed in each household, two people were surveyed: 1) resident aged between 18–40 years old, 2) resident aged 41 years old or above. If there was more than one eligible person available in a household, one participant was selected randomly. Each household in the selected villages was approached. If participants refused to be interviewed or if the resident of the house was not present, it was regarded as a non-response. Trained enumerators administered the questionnaire to the participants. Inclusion criteria for the study were: 1) Orang Asli above 18 years of age and 2) originating from and living in the selected villages.

### Instrument

The questionnaire consists of five main sections: socio-demographic characteristic, type of house and surrounding environment, self-reported prevention practices and control against dengue fever, health beliefs regarding dengue fever and knowledge of dengue fever.

Health beliefs regarding dengue fever was measured using the Health Belief Model (HBM) construct. This construct consists of four mains parts:

1. Perceived Threat consists of two sub-parts which measure the participant’s susceptibility to contracting dengue fever and severity of the seriousness of dengue fever. This was measured on a scale of 1–10, where a higher score indicates higher susceptibility to dengue fever.
2. Perceived Barrier examines the perceptions of barriers to prevent dengue fever among participants. This was also measured on a scale of 1–10, where a higher score indicates higher barriers.
3. Self-efficacy is measured by the behaviour of participants that successfully execute dengue prevention measures. This is measured by a four-point Likert scale that ranges from 1 (strongly agree) to 4 (strongly disagree).
4. Other Contracts and Cue to Actions measures the mosquito problem, frequency of fogging, community participation and other things which effect an individual’s perception which indirectly influences health-related behaviour.

Measurement of knowledge of dengue fever consisted of 43 items divided into five sub-parts: Knowledge about 1) Dengue and the *Aedes* mosquito, 2) Transmission of dengue, 3) Prevention of dengue, 4) Signs and symptoms, and 5) Treatment, curability and precautionary measures for people infected with dengue. For each item, the participants could choose between “yes”, “no”, or “don’t know” responses. For the analyses, correct responses were scored as 1 and incorrect or “don’t know” responses were scored as 0. Scores ranged from 0–43, where higher scores indicate greater knowledge about dengue fever.

Self-reported prevention practices against dengue fever and control was sub-divided into three parts: prevention practices of mosquito breeding, prevention practices of mosquito bites, and prevention practices of dengue transmission. The questions were assessed using nine-item, seven-item, and one-item questions respectively. The options for dengue prevention practices were “not at all”, “rarely”, “sometimes”, “often”, and “not applicable” and were assigned points of 0, 1, 2, 3, and 0 respectively and calculated based on the number of applicable answers. Scores were calculated based on “Number of points obtained” over “Total points of applicable answer”. Results were reported as percentages, where higher percentages indicate higher dengue preventive practices.

This questionnaire was adapted from “Community knowledge, health beliefs, practices and experiences related to dengue fever and its association with dengue prevalence” by Wong et al., 2014. The Cronbach’s alpha >0.70 was reported. Cronbach’s alpha coefficient measurement for prevention of mosquito breeding and mosquito bite were 0.791 and 0.898 respectively, demonstrating good internal consistency. Cronbach’s alpha coefficient measurement for dengue knowledge was 0.916, showing high internal consistency. The Cronbach’s alpha coefficient measurement for self-reported preventive practices in this study was 0.655, demonstrating a good internal consistency.

### Ethics statement and consideration

The study received special permission from the Department of Orang Asli Development (JAKOA) and was approved by the Medical Ethics Committee of the University Malaya Medical Centre, Kuala Lumpur (MEC Ref. No: 896.15). Due to cultural reasons and the sensitivity to outside visitors of the Orang Asli community, a JAKOA representative who was known to the Orang Asli community was present to help during the entire study in the selected villages. Care was taken to safeguard all information from participants who agreed to participate in the study which was voluntary. Informed written and signed consent was obtained prior to beginning the interview.

### Statistical analyses

Other than descriptive analyses, the data were tested for significant relationship between the associative variables and the outcome variables using chi-square test, where P = < 0.05.

The dependent variable (Percentage Scores of Dengue Prevention Practices) was associated to the independent variable (socio-demographic characteristics, HBM construct, cues-to-action and knowledge score) using crosstab and chi-square analysis to see how the variables were associated with dengue prevention practices. Logistic multivariate regression models were used to see the independent effect of each of these variables on the dependent variables. In the modelling strategy, the independent variables were included if they had a p<0.05 on univariate analysis. Associations were expressed with adjusted odds ratios of 95% confidence intervals for each variable included in the multivariate model.

All statistical analyses were performed using SPSS 20.0 (SPSS Inc., Chicago, IL). In all analyses, a p-value of less than 0.05 was considered statistically significant.

## Results

Among the 560 Orang Asli approached from eight states in Peninsular Malaysia, a total of 505 complete responses were obtained with 90.1% response rate. Orang Asli participants who could not comprehend Bahasa Melayu were excluded in this study (n = 3). The survey was conducted between 14 April 2012 and 5 February 2013.

### Socio-demographic characteristics

The study participants consisted of 67.9% (n = 343) female and 32.1% (n = 162) male participants. A majority of the participants were aged between 18–40 years old (n = 366, 72.5%) and only 27.5% (n = 139) of the participants were more than 41 years old of age. Approximately, 36.0% (n = 182) of the participants were from the Temuan tribe. A majority of the participants were living in the forest fringe areas (n = 319, 63.2%), and a minority of the participants were living deep within the jungle (n = 186, 36.8%). Less than half of the participants were primary school educated (n = 205, 40.9%) and most of them were unemployed (n = 253, 50.1%). About 38.4% of the participants living deep within the jungle had no formal education (n = 58). A majority of the participants (n = 347, 68.7%) have less than RM500 as an average monthly household income as they work in the village as a helping hand and do odd jobs around the village. Only 31.3% (n = 158,) of the villagers have an average monthly income of RM500–RM1200 as the participants work as assistant kindergarten teachers in the village and as bus or tourist drivers.

About 39.0% (n = 197) of the Orang Asli participants reported low density of plants and vegetation surrounding their houses and most of them live in the forest fringe (n = 133, 67.5%). Slightly less than half of the participants (n = 252, 49.9%) reported that the density of mosquitoes in their neighbourhood was severe. One third of the participants (n = 173, 34.3%) reported that the authorities fog their village occasionally with insecticide. Only a minority of the participants (n = 60, 11.9%) reported that their village was fogged often, while a majority of the participants reported that their village in the forest fringe was fogged occasionally (n = 136, 78.6%, P = 0.001).

In the self-reported survey among 505 Orang Asli participants, only 2.8% (n = 14) of the participants have had dengue fever. Among the 14 participants, only 85.7% (n = 12) of the participants have been hospitalized for dengue fever (Table 1).

**Table 1:** Socio demographic characteristics differences of Orang Asli aborigine’s knowledge and practices about dengue fever in Peninsular Malaysia.

*Socio Demographic*	Frequency	Knowledge score			Practices Practiced by %		
		0–18	19–36	P	0–50%	51–100%	P
	N (%)	N (%)	N (%)		N (%)	N (%)	
***Gender***							
Male	162 (32.1)	82 (50.6)	80 (49.4)	0.873	72 (44.4)	90 (55.6)	0.973
Female	343 (67.9)	171 (49.9)	172 (50.1)		153 (44.6)	190 (55.4)	
***Age***							
18–40	366 (72.5)	185 (50.5)	181 (49.5)	0.744	154 (42.1)	212 (57.9)	0.069
>41 years	139 (27.5)	68 (48.9)	71 (51.1)		71 (51.1)	68 (48.9)	
***Marital Status***							
Married	441 (87.3)	218 (86.2)	223 (88.5)	0.432	195 (44.2)	246 (55.8)	0.689
Not married	64 (12.7)	35 (13.8)	29 (11.5)		30 (46.9)	34 (53.1)	
***Religion***							
Islam	147 (29.1)	96 (65.3)	51 (34.7)	0.001*	64 (43.5)	83 (56.5)	0.103
Christian	51 (10.1)	27 (52.9)	24 (47.1)		16 (31.4)	35 (68.6)	
Other (Atheist)	307 (60.8)	130 (42.3)	177 (57.7)		145 (47.2)	162 (52.8)	
***States***							
Johor	42 (8.3)	23 (54.8)	19 (45.2)	0.001**	33 (78.6)	9 (21.4)	0.001**
Pahang	57 (11.3)	20 (35.1)	37 (64.9)		22 (38.6)	35 (61.4)	
Negeri Sembilan	89 (17.6)	33 (37.1)	56 (62.9)		43 (48.3)	46 (51.7)	
Kelantan	74 (14.7)	45 (60.8)	29 (39.2)		25 (33.8)	49 (66.2)	
Selangor	48 (9.5)	16 (33.3)	32 (66.7)		27 (56.2)	21 (43.8)	
Melaka	49 (9.7)	16 (32.7)	33 (67.3)		29 (59.2)	20 (40.8)	
Perak	92 (18.2)	52 (56.5)	40 (43.5)		28 (30.4)	64 (69.6)	
Kedah	54 (10.7)	48 (88.9)	6 (11.1)		18 (33.3)	36 (66.7)	
***Tribes***							
Temiar	75 (14.9)	48 (64.0)	27 (36.0)	0.001**	28 (37.3)	47 (62.7)	0.001**
SemoqBeri	32 (6.3)	9 (28.1)	23 (71.9)		14 (43.8)	18 (56.2)	
Semai	90 (17.8)	49 (54.4)	41 (45.6)		26 (28.9)	64 (71.1)	
Temuan	182 (36.0)	65 (35.7)	117 (64.3)		98 (53.8)	84 (46.2)	
Jakun	38 (7.5)	22 (57.9)	16 (42.1)		30 (78.9)	8 (21.1)	
Jah Hut	25 (5.0)	12 (48.0)	13 (52.0)		8 (32.0)	17 (68.0)	
Kensui	47 (9.3)	42 (89.4)	5 (10.6)		15 (31.9	32 (68.1)	
Others	16 (3.2)	6 (37.5)	10 (62.5)		6 (37.5)	10 (62.5)	
**Living Condition**							
Forest fringe	319 (63.2)	158 (49.5)	161 (50.5)	0.738	137 (42.9)	182 (57.1)	0.341
Deep within	186 (36.8)	95(51.1)	91 (48.9)		88 (47.3)	98 (52.7)	
***Highest Education Attainment***							
No Formal Education	151 (29.9)	88 (58.3)	63 (41.7)	0.053	79 (52.3)	72 (47.7)	0.068
Primary Education	205 (40.9)	97 (47.3)	108 (52.7)		83 (40.5)	122 (59.5)	
Secondary Education and more	149 (29.5)	68 (46.3)	81 (54.4)		63 (42.3)	86 (57.7)	
***Occupation***							
Skilled worker	20 (4.0)	4 (20.0)	16 (80.0)	0.010*	9 (45.0)	11 (55.0)	0.831
Non-skilled worker	232 (45.9)	112 (48.3)	120 (51.7)		100 (43.1)	132 (56.9)	
Unemployed(Student,Housewife,Retiree)	253 (50.1)	137 (54.2)	116 (45.8)		116 (45.8)	137 (54.2)	
***Average monthly household income*** ^‡^							
<RM500	347 (68.7)	191 (55.0)	156 (45.0)	0.001**	155 (44.7)	192 (55.3)	0.939
RM500-RM1200	158 (31.3)	62 (39.2)	96 (60.8)		70 (44.3)	88 (55.7)	
***SELF REPORTED—HOUSE &SURROUNDING***							
***Dengue Experience***							
Yes (Once)	14 (2.8)	4 (28.6)	10 (71.4)	0.102	4 (28.6)	10 (71.4)	0.222
No	491 (97.2)	249 (50.7)	242 (49.3)		221 (45.0)	270 (55.0)	
***Type of house***							
Village house (Wood)	241 (47.7)	104 (43.2)	137 (56.8)	0.003**	106 (44.0)	135 (56.0)	0.805
Single story house (PPRT)	264 (52.3)	149 (56.4)	115 (43.6)		119 (45.1)	145 (54.9)	
***Density of vegetation/plants***							
None	22 (4.4)	13 (59.1)	9 (40.9)	0.476	6 (27.3)	16 (72.7)	0.197
Low	197 (39.0)	103 (52.3)	94 (47.7)		96 (48.7)	101 (51.3)	
Moderate	180 (35.6)	90 (50.0)	90 (50.0)		75 (41.7)	105 (58.3)	
A Lot	106 (21.0)	47 (44.3)	59 (55.7)		48 (45.3)	58 (54.7)	
***Fogging Frequency***							
None	86 (17.0)	40 (46.5)	46 (53.5)	0.138	42 (48.8)	44 (51.2)	0.001**
Rarely	186 (36.8)	87 (46.8)	99 (53.2)		101 (54.3)	85 (45.7)	
Occasionally	173 (34.3)	88 (50.9)	85 (49.1)		62 (35.8)	111 (64.2)	
Often	60 (11.9)	38 (63.3)	22 (36.7)		20 (33.3)	40 (66.7)	
***CUES-TO-ACTION***							
***Density of mosquito in neighborhood***							
None	10 (2.0)	7 (70.0)	3 (30.0)	0.446	6 (60.0)	4 (40.0)	0.708
Low	63 (12.5)	32 (50.8)	31 (49.2)		29 (46.0)	34 (54.0)	
Moderate	180 (35.6)	84 (46.7)	96 (53.3)		82 (45.6)	98 (54.4)	
Severe	252 (49.9)	130 (51.6)	122 (48.4)		108 (42.9)	144 (57.1)	
**Health Belief Modal (HBM)**							
**Perceived Severity**							
1–5	60 (11.9)	33 (55.0)	27 (45.0)	0.419	29 (48.3)	31 (51.7)	0.530
6–10	445 (88.1)	220 (49.4)	225 (50.6)		196 (44.0)	249 (56.0)	
**Perceived Susceptibility**							
1–5	414 (82.0)	219 (52.9)	195 (47.1)	0.007**	198 (47.8)	216 (52.2)	0.002**
6–10	91 (18.0)	34 (37.4)	57 (62.6)		27 (29.7)	64 (70.3)	
**Perceived Barriers**							
1–5	435 (86.1)	218 (50.1)	217 (49.9)	0.986	184 (42.3)	251 (57.7)	0.011**
6–10	70 (13.9)	35 (50.0)	35 (50.0)		41 (58.6)	29 (41.4)	
**Self-Efficacy**							
Agree	129 (25.5)	44 (34.1)	85 (65.9)	0.001**	64 (49.6)	65 (50.4)	0.180
Disagree	376 (74.5)	209 (55.6)	167 (44.4)		161 (42.8)	215 (57.2)	
**Knowledge Score**							
0–18	253 (50.1)				126 (49.8)	127 (50.2)	0.017**
19–36	252 (49.9)				99 (39.3)	153 (60.7)	

^‡^ 1 US Dollar = 3.8 Malaysian Ringgit (MYR)

***Association is significant at the 0.001 level.

**Association is significant at the 0.01 level.

*Association is significant at the 0.05 level.

### Knowledge

Table 2 shows that 85.9% (n = 434) of the participants correctly answered that dengue fever is transmitted by a mosquito. Most of them (n = 324, 64.2%) know that dengue fever is caused by the *Aedes* mosquito. Only a minority of the participants (n = 89, 17.6%) know that the *Aedes* mosquitoes do not live in places with a lot of plants and 39.8% (n = 201) know that dengue is a virus. Slightly more than half of the participants know that dengue fever can spread by an *Aedes* mosquito biting an infected person and then biting another (n = 296, 58.6%).

**Table 2:** Overall knowledge about dengue fever (correct responses).

Overall Knowledge for dengue fever (0–43)	Frequency (N)	%
Mean knowledge score (SD)	18.41 (9.45)	
0–18	253	50.1
19–36	252	49.9
**Knowledge about dengue and Aedes mosquito**		
Transmission by mosquito	434	85.9
Virus transmitted by Aedes mosquito	324	64.2
Dengue fever is a virus	201	39.8
Dengue Fever may become Dengue Haemorrhagic fever	320	63.4
Dengue Haemorrhagic fever can be fatal	331	65.5
Dengue Haemorrhagic fever usually occur to people who have had several dengue infection	185	36.6
Aedes mosquito has black and white stripes on its leg and body	319	63.2
Breeds in clean stagnant water	246	48.7
Lives in houses and building rather than natural wetlands	276	54.7
Do not live in places with lots of plants	89	17.6
Bite during dusk and dawn	311	61.6
Aedes mosquito biting an infected person can spread it to another	296	58.6
Dengue fever usually appear 4–7 days after being bitten	200	39.6
Dengue fever can be transmitted by blood	240	47.5
Aedes mosquito egg can contain dengue virus	224	44.4
A person can only get dengue once	225	44.6
Dengue epidemic occurs only during rainy season	252	49.9
**Knowledge about the transmission of dengue**		
Aedes mosquito biting an infected person can spread to another	296	58.6
Fever after 4–7days after mosquito bite	200	39.6
Transmitted by touch	192	38.0
Transmitted by air	184	36.4
Transmitted by body fluids	174	34.5
Transmitted by blood	241	47.5
The mosquito egg contains dengue virus	224	44.4
Only can get dengue fever once	225	44.6
Dengue epidemic occurs any season	252	49.9
**Knowledge about prevention**		
Weekly change of stagnant water (pet bowls, vases)	410	81.2
Put Abate/chemical in water containers	274	54.3
Covering water containers	416	82.4
Emptying or drying out containers around the house	424	84.0
Proper disposal of items that can retain water	431	85.3
**Sign and Symptoms**		
5–6 days high fever	312	61.8
Chills	288	57.0
Rash	223	44.2
Pain in the eyes	190	37.6
Joint pains	226	44.8
Headache	246	48.7
Stomach ache	173	34.3
Nausea and vomiting	179	35.4
**Knowledge about treatment, curability and precaution measures for people infected with dengue fever**		
**No Medication for dengue**		
Yes	134	26.5
No	233	46.1
Do not know	138	27.3
**Immediate treatment can prevent complication and death**		
Yes	296	58.6
No	111	22.0
Do not know	98	19.4
**There is vaccine to prevent dengue**		
Yes	171	33.9
No	126	25.0
Do not know	197	39.0

The mean total knowledge score for the overall sample was 18.4, (SD±9.45) out of a possible score of 43. In Table 1, slightly more than half of the participants, 50.1% (n = 253) had a range of total dengue knowledge score of 0 to 18, while 49.9% of the participants (n = 252) had a range of total dengue knowledge score of 19 to 36. Slightly more than half of the participants (n = 161, 50.5%) living in the forest fringe had a range of total dengue knowledge score of 19 to 36, while 51.1% of the participants living deep within the jungle (n = 95) had a range of total dengue knowledge score of 0 to 18. Participants with more than RM500 average monthly income had a range of total dengue knowledge score of 19 to 36 compared to those who earn RM500–RM1200 a month (n = 96, 60.8%). Participants living in village houses (n = 137, 56.8%) had a range of dengue knowledge score of 19 to 36, compared to participants living in single story houses (n = 115, 43.6%) under the Housing Project for the Hardcore Poor (PPRT).

As shown in Table 1, the dengue knowledge score was significantly different between religions, tribes, occupations, average monthly incomes, and types of house. The Temuan tribe had a range of knowledge score of 19 to 36 compared to the other tribes investigated (n = 64, 71.1%). Almost 80% of the skilled workers (n = 16) had a range of dengue knowledge score of 19 to 36 compared to the unskilled (n = 120, 51.7%) and unemployed (n = 116, 45.8%). The proportion of dengue knowledge score range of 19 to 36 was significantly higher among Orang Asli participants with higher perceived susceptibility of dengue (level of susceptibility 6–10) (n = 57, 62.6%, P = 0.007) than participants with lower perceived susceptibility of dengue (level of susceptibility 1–5). It was found that the proportion of dengue knowledge score range of 19 to 36 was significantly higher in those who agreed on self-efficiency in dengue prevention than those who disagreed on self-efficiency in the prevention of dengue in the chi square test (n = 85, 65.9%, P = 0.001).

### Dengue prevention practices

In this study, more than half of the participants (n = 280, 55.4%) had a total dengue prevention practices percentage score range of 51 to 100. Table 3 shows that most of the participants (n = 491, 97.2%) practiced proper disposal of items that can collect rain water. Most of them also practiced proper disposal of household garbage (n = 479, 94.9%) and clearing out of the debris that blocked water flow (n = 476, 94.3%). It was also noted that 93.9% (n = 474) of the participants practice cleaning the areas surrounding their house frequently as one of the reasons to prevent dengue. Only a minority of the participants (n = 93, 18.4%) use Abate or chemicals in water storage containers to prevent dengue mosquito breeding, and 10.7% (n = 54) of the participants use mosquito repellent on their body to prevent mosquito bites.

**Table 3:** Overall practices practiced by the Orang Asli to prevent dengue fever.

Overall Practice for dengue fever	Frequency (N)	%
**Prevention of mosquito breeding**		
Cover all water used for storing in or outside the house	393	77.8
Change stored water in flower vases, drip tray or pails	409	81.0
Put abate or chemical in water storage containers	93	18.4
Examine for mosquito larvae in containers for storing water	263	52.1
Clear out debris that may block water flow in drain or roof gutters	476	94.3
Proper disposal of items that can collect rain water	491	97.2
**Cue to action**		
Proper disposal of household garbage	479	94.9
Clean up surrounding house area	474	93.9
Take mosquito preventive measure before going on long holiday	336	66.5
**Prevention of mosquito bite**		
Sleep in mosquito net of have mosquito screens on windows	392	77.6
Use mosquito coil, electrical mosquito mat, liquid vapourizer	269	53.3
Spraying dark places with an insecticidal spray	187	37.0
Use mosquito repellent on body	54	10.7
Avoid dark areas in the home where there is no light and no wind	179	35.4
Wear long-sleeved shirts and pants to avoid mosquito bites	272	53.9
Wear bright colour clothes to avoid mosquito bites	182	36.0
**Prevention of dengue transmission**		
Take measure to prevent mosquito from biting a dengue patient	251	49.7

The total percentage scores of dengue prevention practices were significantly different among the socio-demographic variables and self-reported variables, specifically the Orang Asli tribes and fogging frequency. As shown in Table 1, the Semai tribe reported a range of percentage scores of 51–100 for dengue prevention practices (n = 64, 71.1%) compared to the other tribes investigated. A significantly higher proportion of participants reported that their village was often fogged had a range of percentage scores of 51–100 for dengue prevention practices (n = 40, 66.7%). The proportion of dengue prevention practices percentage scores in the range of 51–100 was significantly higher among participants with higher perceived susceptibility to dengue fever (level of susceptibility 6–10) (n = 64, 70.3%) compared to participants with lower perceived susceptibility to dengue fever (level of susceptibility 1–5). A significantly higher proportion of participants with a higher perceived barrier to prevention of dengue (level of barriers 6–10) had a range of percentage scores of 0–50 for dengue prevention practices (n = 41, 58.6%) compared to participants with lower perceived barriers to prevention of dengue (level of barriers 1–5) (n = 184, 42.3%). It was also found that participants with a range of total dengue knowledge score of 19–36 had significant dengue prevention practices percentage score range of 51–100 (n = 153, 60.7%).

In Table 4, the multiple logistic regressions indicated that participants with a lower dengue knowledge score (score of 0–18) were less likely (OR = 0.63, 95%CI = 0.44–0.92, P = 0.015) to perform dengue prevention practices compared to the reference group (participants with a higher dengue knowledge score of 19–36). The results also indicated that the two main constructs of HBM (perceived susceptibility and perceived barriers) were significant correlates of dengue prevention practices. Participants with lower perceived susceptibility (level of susceptibility 1–5) was less likely (OR = 0.54, 95%CI = 0.33–0.89, P = 0.018) to perform dengue prevention practices compared with those with the reference level of susceptibility (level 6–10). Likewise, those with lower perceived barriers (level 1–5) had a higher likelihood (OR = 2.06, 95%CI = 1.21–3.53, P = 0.008) to perform dengue prevention practices compared with those with the reference level of barriers (level 6–10). Orang Asli villages that were not fogged (OR = 0.49, 95%CI = 0.24–0.99, P = 0.045) or rarely fogged (OR = 0.40, 95%CI = 0.22–0.75, P = 0.004) had a lower likelihood to perform dengue prevention practices when compared with the villages that were often fogged (reference group).

**Table 4:** Results of logistic regression analyses predicting practices practiced to prevent dengue fever.

*Socio Demographic*	Frequency	Practices Practiced by %			Multiple Logistic Regression		
		0–50%	51–100%	P	β	B (CI)	P
	N (%)	N (%)	N (%)				
***Fogging Frequency***							
None	86 (17.0)	42 (48.8)	44 (51.2)	0.001**	-0.716	0.49 (0.24–0.99)	0.045
Rarely	186 (36.8)	101 (54.3)	85 (45.7)		-0.911	0.40 (0.22–0.75)	0.004**
Occasionally	173 (34.3)	62 (35.8)	111 (64.2)		-0.131	0.88 (0.47–1.65)	0.685
Often	60 (11.9)	20 (33.3)	40 (66.7)			Reference	
**Health Belief Modal (HBM)**							
**Perceived Susceptibility**							
1–5	414 (82.0)	198 (47.8)	216 (52.2)	0.002**	-0.611	0.54 (0.33–0.89)	0.018*
6–10	91 (18.0)	27 (29.7)	64 (70.3)			Reference	
**Perceived Barriers**							
1–5	435 (86.1)	184 (42.3)	251 (57.7)	0.011*	0.724	2.06 (1.21–3.52)	0.008**
6–10	70 (13.9)	41 (58.6)	29 (41.4)			Reference	
**Knowledge Score**							
0–18	253 (50.1)	126 (49.8)	127 (50.2)	0.017**	-0.458	0.63 (0.44–0.92)	0.015*
19–36	252 (49.9)	99 (39.3)	153 (60.7)			Reference	

***Association is significant at the 0.001 level.

**Association is significant at the 0.01 level.

*Association is significant at the 0.05 level

Logistic regression model; Hosmer and Lemeshow test, χ^2^ (7) = 4.767, P = 0.688; Cox & Snell R^2^ = 0.071; Nagelkerke R^2^ = 0.096

## Discussion

### Self-reported dengue experience

In Malaysia, the actual number of dengue cases is higher than the number of self-reported dengue cases [2]. In a recent study conducted in Malaysia among the Orang Asli, the Semai Perak community showed the highest prevalence of dengue fever (> 50%). However, in this study, only a minority of the Orang Asli participants reported that they have had dengue fever. The low self-report of dengue experience in this study may indicate that the Orang Asli were not aware that they have had dengue fever. This may imply that they would have had dengue fever and recovered from it without receiving treatment. Hence, it is important to educate the Orang Asli to distinguish the disease and encourage the Orang Asli to visit a health care practitioner to avoid further complication and fatalities due to dengue fever.

### Social demographic association with dengue knowledge

#### Occupation and average monthly income

The occupation group with highest knowledge scores was the skilled workers. Most of them are higher educated, thus have higher dengue knowledge score compared to those non skilled workers and those unemployed. Further, most of the skill workers were men working outside of the village and are exposed to information of dengue through mass media outside their villages [31]. In contrast, those who are unemployed and non-skilled workers are mainly housewives. Secondly, this study also found those who had an average month income of RM500-RM1200 had a significantly higher knowledge score, most of which were skilled workers that sought employment outside their villages. This study implies lack of dengue awareness campaign that target on the orang Asli communities. Therefore, dengue awareness educational intervention should be targeted among the Orang Asli community and also the Orang Asli that live deep within the jungle.

#### Living condition

In this study, participants living deep within the jungle had a low total knowledge score compared to those living in the forest fringe. Orang Asli participants from Kedah living deep within the jungle had a lower total score of knowledge as most of them were living in remote areas where resources was not readily available. A study conducted among the rural and urban population in India showed that the urban population had sufficient knowledge on dengue compared to those living in rural areas [32]. This may imply a lack of dengue prevention campaign outreach among the Orang Asli community living in remote areas. In Malaysia, the majority of dengue educational messages were in the form of mass media such as television and radio advertisements [31]. As Orang Asli live in remote areas, they may not be exposed to such educational messages. An educational intervention should be provided focusing on enhancing knowledge about dengue among the Orang Asli.

#### Type of house

In this study, the type of house was significantly associated with dengue knowledge score. Participants that lived in village houses had a higher dengue knowledge score compared to those living in single story houses (PPRT). In a study conducted in Southern Thailand, houses constructed with corrugated iron sheets were significantly associated with secondary dengue infections [33]. The temperature in a house can affect the incubation period of the mosquito promoting a quicker transmission of the virus compared to houses constructed with other materials. Today, with the help of the government and due to mass development in Malaysia, the government is helping the Orang Asli Aborigine to migrate out of the jungle by providing PPRT houses and also work near the forest-fringe areas where it is accessible to school and employment. In addition to climatic changes, particularly housing may affect vector abundance and disease transmission [34]. Thus, the general construction of house and primary education is important in predicting dengue fever.

### Participant’s knowledge on dengue—sign and symptoms

The participants in this study had a low overall mean knowledge score of 18.4 (out of a possible highest score of 43). Most of the participants were aware that dengue is transmitted by a mosquito and may evidently portray as fever 4–7 days after a mosquito bite. This is in accordance to a study conducted in Jamaica and India, where participants could identify one of the common and obvious sign and symptom is portray of fever [25, 35]. However, they had low knowledge about joint pains, rashes, and headaches which are the main sign and symptoms of dengue fever. This implies that the majority of the participants did not have good knowledge about the signs and symptoms of dengue fever. Therefore, it is vital to educate the Orang Asli on the signs and symptoms of dengue fever so as to seek immediate treatment to prevent unwarranted death caused by dengue fever.

### Knowledge about dengue prevention

The ideal approach to prevent dengue is to eliminate areas where the dengue mosquito lays its eggs. This study found that the majority of the participants had adequate knowledge of proper disposal of items that can retain water and proper disposal of garbage, which leads to prevention of dengue fever. Cleaning those surroundings of the house which can bring about stagnant water is basic to keeping dengue mosquito from spreading. However, the majority of the participants did not know that the dengue causing mosquito breeds in clean water. Participants presume that it breeds in dirty water, as mostly dengue mosquito breeds outdoors in stagnant water in drains or empty cans which are dirty. The majority of the public associate “dirty” sites outside the house as prominent breeding sites for dengue causing mosquitoes [36]. There appears to be a misconception that dengue mosquitos only breed in dirty water. This implies education should focus on informing the Orang Asli community that in fact dengue mosquitoes prefer to breed in clean stagnant water such as in water jars and flower pots.

### Health beliefs with regards to dengue prevention practices

The perceived susceptibility and perceived barriers constructs of the HBM were significantly associated with prevention practices. Participants with lower perceived susceptibility to dengue fever were less likely to carry out prevention practices against dengue fever. This could be because the majority of the Orang Asli are not aware of the dangers of dengue fever and have not experienced it for themselves. Other studies have shown that if action is likely to occur, the individual perceives the susceptibility of getting the illness [37]. Education programmes need to highlight the risk of getting the disease to create awareness among people who are unconscious of the serious threat of dengue. Testimonials and campaigns can be used from families who have lost a family member due to dengue fever.

In this study, participants with lower perceived barriers were significantly associated with higher dengue prevention practices. In order to effectively perform dengue prevention, it is imperative to remove barriers that impede taking action against dengue. Among the barriers are i) low perceived susceptibility, ii) lack of self-efficacy, iii) unsure perceived susceptibility, and iv) lack of perceived benefit. Authorities should provide facilities to remove these barriers such as increasing community participation to eliminate mosquito breeding sites and increase campaigns to boost responsibilities towards neighbourhood cleanliness to facilitate prevention practices among the Orang Asli communities.

### Dengue prevention practices

It was observed that slightly more than half of the participants took precautions against mosquito bites by using mosquito coils. Participants need to be aware that using mosquito coils should also be practiced at home to prevent mosquito bites as the dengue mosquito can be found everywhere, not only outdoors. In a study conducted by Dieng H. et al. (2010) it was found that indoor containers contained immature Aedes mosquito eggs which further shows that the Aedes mosquitoes have adapted to breeding indoors due to easy access to a blood source. Therefore, precautions against mosquito bites to prevent dengue fever from spreading should not only be taken outdoors, but in-house prevention is also important. Most of the Orang Asli live below the poverty line, and cannot afford to buy precautionary materials such as mosquito coils or bed nets. Therefore, it is recommended that the government should put more emphasis on introducing cost effective ways of preventing mosquitoes and dengue fever.

Only a minority of the Orang Asli were aware of the use of Abate which can prevent mosquito breeding in water containers. Using Abate was found useful in Thailand to reduce *Aedes aegypti* in water holding containers [38]. The reasons for lack of Abate use among the Orang Asli is due to lack of awareness of Abate in dengue prevention. Another reason is, the Orang Asli are unable to obtain Abate easily as they have to travel into the nearest towns to obtain Abate for their use. A large number of the Orang Asli living in remote areas do not have proper water supply, and therefore mostly depend on containers to store water. Thus, the Orang Asli communities should be educated about Abate in dengue prevention and that the use of Abate is not dangerous to health.

### Multivariate analysis

The findings from the multivariate analysis found the highest odds of dengue prevention practice among the following: dengue knowledge level, perceived barriers to performing dengue prevention, fogging frequency, and perceived susceptibility to dengue fever. Low knowledge on symptoms and prevention of dengue leads to poorer precautionary practices against dengue fever. Therefore, this study highlights that health education, campaigns, and knowledge of dengue fever is highly needed and necessary in order to boost preventive practices among the Orang Asli community.

The multiple logistic regression analysis also found a significant association between fogging frequency in the villages and prevention practices. Fogging is a commonly used method in dengue prevention in many countries [39]. One study proved that fogging has greatly influenced the reduction of dengue cases but is influenced by seasonality and the level of transmission intensity of dengue fever in an area [13]. In Malaysia, fogging is commonly conducted when dengue fever is highly reported in the area. It is likely that fogging creates higher awareness among the community thus triggers higher prevention practices. Therefore, this may imply that fogging may be beneficial both in eradicating adults mosquitoes as well as create awareness and enhance dengue prevention practises among the targeted communities.

### Limitations of the study

The study had a few limitations. Orang Asli villages were selected based on accessibility by land transport. This may result in selection bias because of the sample which was not representative of the overall Orang Asli population in Peninsular Malaysia since the Orang Asli living in more remote or inaccessible areas were not surveyed. All information obtained from the interview was self-reported, thus bias towards socially desirable responses and behaviours might exist.

Despite some of the limitations in the study, the results provided useful outcomes and knowledge that would guide government officials in the development of programmes and activities to initiate dengue prevention to address the every growing problem of dengue fever. More community based projects should be conducted among the Orang Asli tribes to educate them on dengue fever and its fatal disease. One of the main sources of dengue awareness and education is through mass media. Therefore, more advertisements and bill boards should be put up in outskirts and remote areas emphasizing the seriousness of dengue fever.

### Conclusion

From this study, several conclusions could be inferred with important implications for dengue fever prevention practices. Firstly, the findings indicate that the level of knowledge about dengue fever, signs and symptoms, and prevention among the participants was low. Secondly there were significant differences of knowledge scores in different religions, states, occupations, average monthly incomes, and types of house. Thirdly, dengue knowledge level, perceived barriers to perform dengue prevention, fogging frequency, and perceived susceptibility to dengue fever were significant factors associated with dengue prevention practices. Therefore, educational and health programmes should focus on enhancing dengue knowledge and perceived susceptibility of acquiring dengue and reducing perceived barriers to perform dengue prevention practices among the Orang Asli communities in Malaysia. Mosquito fogging may create awareness among Orang Asli and enhance their dengue prevention practices. Therefore, less fogged areas need to be the focus of education interventions. Awareness of dengue should be adhered to more enthusiastically within the community and to enhance health beliefs.

## Supporting Information

S1 Checklist STROBE checklist of items that should be included in reports of cross-sectional studies. (DOC)
