# Supplementary material for: Practices of Dengue Fever Prevention and the Associated Factors among the Orang Asli in Peninsular Malaysia
Source: PLoS Negl Trop Dis. 2015 Aug 12;9(8):e0003954. doi: 10.1371/journal.pntd.0003954 (PMC4534093; doi:10.1371/journal.pntd.0003954)
Supplement: S1 Checklist — (DOC) [file pntd.0003954.s001.doc]

STROBE Statement—Checklist of items that should be included in reports of ***cross-sectional studies***

|  | Item No | Recommendation |
| --- | --- | --- |
| **Title and abstract** | 1 | (*a*) Indicate the study’s design with a commonly used term in the title or the abstract  Added in Abstract  A cross-sectional survey was conducted in 16 randomly selected Orang Asli villages from eight states in Peninsula Malaysia…….. |
|  |  |  |
|  |  | (*b*) Provide in the abstract an informative and balanced summary of what was done and what was found  Information provided |
| Introduction | | |
| Background/rationale | 2 | Explain the scientific background and rationale for the investigation being reported  Stated in manuscript |
| Objectives | 3 | State specific objectives, including any prespecified hypotheses  Stated in manuscript |
| Methods | | |
| Study design | 4 | Present key elements of study design early in the paper  A cross-sectional study was performed in each household, two people were surveyed…… |
| Setting | 5 | Describe the setting, locations, and relevant dates, including periods of recruitment, exposure, follow-up, and data collection  Described in manuscript |
| Participants | 6 | (*a*) Give the eligibility criteria, and the sources and methods of selection of participants  (Page 6)  A cross-sectional study was performed in each household, two people were surveyed: 1) resident aged between 18–40 years old, 2) resident aged 41 years old or above. If there was more than one eligible person available in a household, one participant was selected randomly. Each household in the selected villages was approached. If participants refused to be interviewed or if the resident of the house was not present, it was regarded as a non-response. Trained enumerators administered the questionnaire to the participants. Inclusion criteria for the study were: 1) Orang Asli above 18 years of age and 2) originating from and living in the selected villages. |
| Variables | 7 | Clearly define all outcomes, exposures, predictors, potential confounders, and effect modifiers. Give diagnostic criteria, if applicable  (Page 6) |
| Data sources/ measurement | 8* | For each variable of interest, give sources of data and details of methods of assessment (measurement). Describe comparability of assessment methods if there is more than one group  Clearly stated in Page 7 |
| Bias | 9 | Describe any efforts to address potential sources of bias  The research group approached Orang Asli members with JAKOA's supervision to acquire better acknowledgement and responses. |
| Study size | 10 | Explain how the study size was arrived at  Sample size not calculated, all the community in the selected villages were approached |
| Quantitative variables | 11 | Explain how quantitative variables were handled in the analyses. If applicable, describe which groupings were chosen and why |
| Statistical methods | 12 | (*a*) Describe all statistical methods, including those used to control for confounding |
|  |  | (*b*) Describe any methods used to examine subgroups and interactions |
|  |  | (*c*) Explain how missing data were addressed  Missing data or incomplete responses were not included in the analyses |
|  |  | (*d*) If applicable, describe analytical methods taking account of sampling strategy |
|  |  | (*e*) Describe any sensitivity analyses |
| Results | | |
| Participants | 13* | (a) Report numbers of individuals at each stage of study—eg numbers potentially eligible, examined for eligibility, confirmed eligible, included in the study, completing follow-up, and analysed  Page 10 |
|  |  | (b) Give reasons for non-participation at each stage  Orang Asli participants who could not comprehend Bahasa Melayu were excluded in this study (n=3). |
|  |  | (c) Consider use of a flow diagram |
| Descriptive data | 14* | (a) Give characteristics of study participants (eg demographic, clinical, social) and information on exposures and potential confounders  Summarized in Table 1 |
|  |  | (b) Indicate number of participants with missing data for each variable of interest  Among the 560 Orang Asli approached from eight states in Peninsular Malaysia, a total of 505 complete responses were obtained with 90.1% response rate |
| Outcome data | 15* | Report numbers of outcome events or summary measures  (Table 1) |
| Main results | 16 | (*a*) Give unadjusted estimates and, if applicable, confounder-adjusted estimates and their precision (eg, 95% confidence interval). Make clear which confounders were adjusted for and why they were included (Table 1) |
|  |  | (*b*) Report category boundaries when continuous variables were categorized |
|  |  | (*c*) If relevant, consider translating estimates of relative risk into absolute risk for a meaningful time period |
| Other analyses | 17 | Report other analyses done—eg analyses of subgroups and interactions, and sensitivity analyses |
| Discussion | | |
| Key results | 18 | Summarise key results with reference to study objectives  (Page 14) |
| Limitations | 19 | Discuss limitations of the study, taking into account sources of potential bias or imprecision. Discuss both direction and magnitude of any potential bias  (Page 21)  The study had a few limitations. Orang Asli villages were selected based on accessibility by land transport. This may result in selection bias because of the sample which was not representative of the overall Orang Asli population in Peninsular Malaysia since the Orang Asli living in more remote or inaccessible areas were not surveyed. All information obtained from the interview was self-reported, thus bias towards socially desirable responses and behaviours might exist.  Despite some of the limitations in the study, the results provided useful outcomes and knowledge that would guide government officials in the development of programmes and activities to initiate dengue prevention to address the every growing problem of dengue fever. More community based projects should be conducted among the Orang Asli tribes to educate them on dengue fever and its fatal disease. One of the main sources of dengue awareness and education is through mass media. Therefore, more advertisements and bill boards should be put up in outskirts and remote areas emphasizing the seriousness of dengue fever. |
| Interpretation | 20 | Give a cautious overall interpretation of results considering objectives, limitations, multiplicity of analyses, results from similar studies, and other relevant evidence |
| Generalisability | 21 | Discuss the generalisability (external validity) of the study results |
| Other information | | |
| Funding | 22 | Give the source of funding and the role of the funders for the present study and, if applicable, for the original study on which the present article is based |

*Give information separately for exposed and unexposed groups.

**Note:** An Explanation and Elaboration article discusses each checklist item and gives methodological background and published examples of transparent reporting. The STROBE checklist is best used in conjunction with this article (freely available on the Web sites of PLoS Medicine at http://www.plosmedicine.org/, Annals of Internal Medicine at http://www.annals.org/, and Epidemiology at http://www.epidem.com/). Information on the STROBE Initiative is available at www.strobe-statement.org.
